# TRPA1/M8 agonists upregulate ciliary beating through the pannexin-1 channel in the human nasal mucosa

**DOI:** 10.1007/s11033-022-08201-7

**Published:** 2022-12-21

**Authors:** Thi Nga Nguyen, Yuma Koga, Tetsuro Wakasugi, Takuro Kitamura, Hideaki Suzuki

**Affiliations:** 1 Department of Otorhinolaryngology-Head and Neck Surgery, School of Medicine, University of Occupational and Environmental Health, 1-1 Iseigaoka, Yahatanishi-Ku, Kitakyushu, 807-8555 Japan; 2 Faculty of Public Health, Vinh Medical University, Vinh City, Vietnam

**Keywords:** Ciliary beating, Nasal mucosa, Thermoreceptor, TRPA1, TRPM8, Pannexin-1

## Abstract

**Background:**

Nasal breathing is important for maintaining physiological respiration. However, airflow in the nasal cavity has an inherent cooling effect and may suppress ciliary beating, an essential frontline defense in the airway. Nasal airflow is thought to be perceived by thermoreceptors for cool temperatures. We herein investigated the effect of the activation of thermosensitive transient receptor potentials (TRPs) for cool/cold temperatures on ciliary beating to search for a compensatory mechanism.

**Methods:**

Inferior turbinates were collected from patients with chronic hypertrophic rhinitis. Ex vivo ciliary beat frequency (CBF) and ATP release were measured using a high-speed digital video camera and by luciferin-luciferase assay, respectively. Intracellular Ca^2+^ ([Ca^2+^]_i_) imaging of isolated ciliated cells was performed using Fluo-8. The nasal mucosae were also subjected to fluorescence immunohistochemistry and real-time RT-PCR for TRPA1/TRPM8.

**Results:**

CBF was significantly increased by adding either cinnamaldehyde (TRPA1 agonist) or l-menthol (TRPM8 agonist). This increase was inhibited by pannexin-1 blockers, carbenoxolone and probenecid. Cinnamaldehyde and l-menthol also increased the ATP release from the nasal mucosa and [Ca^2+^]_i_ of isolated ciliated cells. Immunohistochemistry detected TRPA1 and TRPM8 on the epithelial surface including the cilia and in the submucosal nasal glands. Existence of these receptors were confirmed at the transcriptional level by real-time RT-PCR.

**Conclusions:**

These results indicate the stimulatory effect of the activation of TRPA1/TRPM8 on ciliary beating in the nasal mucosa, which would be advantageous to maintain airway mucosal defense against the fall of temperature under normal nasal breathing. This stimulatory effect is likely to be mediated by pannexin-1.

## Introduction

Nasal breathing plays an important role in maintaining physiological respiration. Upper airway inflammatory diseases, such as acute/chronic rhinosinusitis, often block nasal breathing. Nasal obstruction, or lack of sensation of nasal airflow, often causes impaired concentration, drowsiness, fatigue, headache, and sleep disturbance [1, 2]. Airflow in the nasal cavity is thought to be perceived by the transient receptor potential (TRP) M8 channel [3, 4], which is known to be a thermoreceptor for cool temperatures. This channel in the nasal mucosa is activated in response to cooling by inhaled air.

The mucociliary transport function of the respiratory tract, including the sinonasal cavity, has a significant role in the frontline defense of the body; namely, eliminating various airborne foreign materials, allergens and pathogens from the mucosal surface. This function is largely dependent on the ciliary beating of the airway mucosa. Like other biological reactions, ciliary beating may be suppressed as the mucosal temperature falls [5], suggesting that nasal breathing, which cools the mucosa, would be disadvantageous to the mucociliary transport function of the nasal mucosa. However, it would be counterintuitive if nasal breathing would impair the upper airway defense mechanism.

From these observations and knowledge, we speculated that the airway mucosa has some compensatory mechanism to maintain ciliary beating against the fall of temperature. In the present study, we investigated the effect of TRPM8 activation by a specific agonist on ciliary beating of the human nasal mucosa in ex vivo experiments. In addition, we examined the effect of activating TRPA1, another thermoreceptor for cold temperature [6].

## Materials and methods

### Patients and sample collection

A total of 25 patients with chronic hypertrophic rhinitis were enrolled in this study. They were 18 males and 7 females, aged 18–76 years, with an average age of 49.9 years. Total and/or specific serum IgE levels were positive in 16 patients (64.0%). Specific serum IgE levels were measured for house dust mites, Japanese cedar pollen, cypress pollen, orchard grass pollen, short ragweed pollen, timothy grass pollen and *Aspergillus*, which are major airborne allergens in Japan. Eight patients had bronchial asthma. The inferior turbinate bone was resected together with the lateral mucosa of the turbinate under general anesthesia. The collected inferior turbinates were immediately soaked in O_2_-saturated Hank’s balanced salt solution (HBSS; 8000 (in mg/L) NaCl, 400 KCl, 350 NaHCO_3_, 140 CaCl_2_, 100 MgCl_2_^.^6H_2_O, 100 MgSO_4_^.^7H2O, 60 KH_2_PO_4_, 47.8 Na_2_HPO_4_, and 1000 glucose) and thoroughly washed with HBSS to remove surface mucus. The lateral mucosa of the collected turbinate was separated from the underlying bone with surgical scissors and subjected to experimental observation and processing.

### Chemicals

Carbenoxolone (a pannexin-1 blocker) was purchased from Sigma-Aldrich (St. Louis, MO, USA). Cinnamaldehyde (a TRPA1 agonist), l-menthol (a TRPM8 agonist) and probenecid (a pannexin-1 blocker) were obtained from Wako Pure Chemical Industries (Osaka, Japan). Fluo-8 acetoxymethyl ester (Fluo-8 AM) was bought from AAT Bioquest (Sunnyvale, CA, USA). Cinnamaldehyde, l-menthol, probenecid and Fluo-8 AM were each dissolved in DMSO to make a ×1000 concentrated stock solution. Carbenoxolone was dissolved in distilled water to make a ×1000 concentrated stock solution. The final concentration of DMSO was 0–0.2%. Our previous investigation had confirmed that 0.1–0.2% DMSO does not significantly change the baseline ciliary beat frequency (CBF) [7, 8].

### Preparation of mucosal pieces from the turbinate sample for video recording

The turbinate mucosa was cut into slender strips along the mucosal surface in the vertical direction using a razor blade. The mucosal strips were immediately immersed in O_2_-saturated HBSS and transferred into another tube filled with O_2_-saturated HBSS containing chemical(s) to be tested. The sample was then put in a 20×6×1-mm test chamber filled with the same solution containing the chemical(s), and mucociliary movement was observed under a Nikon Eclipse 80i phase-contrast light microscope (Nikon, Tokyo, Japan) equipped with a high-speed digital video camera. All procedures were performed at room temperature (approx. 24 °C) and completed within 3 h after sample collection.

### Measurement of ciliary beat frequency

Four ciliary beat recordings of 2–3 sec each were made at 60-sec intervals at a speed of 200 frames/sec using the high-speed digital imaging system HAS-U1 (DITECT, Tokyo, Japan) and were analyzed by HAS-XViewer Camera Memory ver. 1.2.12 (DITECT). The number of ciliary beats was counted manually by checking the video in a slow replay mode. CBF was measured at three different sites of each mucosal strip. The CBF value in each experiment was determined by averaging the 12 measurements (4 recordings×3 sites).

### Measurement of ATP release from the nasal mucosa

Ex vivo ATP release from the nasal mucosa was measured as described previously [9]. Round pieces measuring 4 mm in diameter were cut out from the turbinate mucosa using a metal circular punch. The cutout mucosal pieces were preincubated in O_2_-saturated HBSS for 30 min. After a brief wash with HBSS, the samples were incubated in a 12-well culture plate containing 4 ml of HBSS in each well with or without a TRP agonist. One hundred μl of medium was then collected by an ATP water-testing device, AquaSnap Total (Hygiena, Camarillo, CA, USA), and the ATP concentration was measured by a luciferin-luciferase assay using a SystemSURE luminometer (Hygiena). A calibration curve was made by measuring the ATP levels of standard ATP solutions (10^−11^–10^−8^ M). All procedures were performed at room temperature (approx. 24 °C) and completed within 1 h after the sample collection.

### ***Intracellular Ca***^***2***+^***imaging of isolated ciliated cells***

Ciliated nasal epithelial cells were isolated by gently brushing the surface of the turbinate mucosa in O_2_-saturated HBSS, and intracellular Ca^2+^ [Ca^2+^]_i_ imaging was performed according to our previous method [10]. Cell suspension was transferred to a thin-bottomed petri dish (Matsunami Glass Ind., Osaka, Japan) coated with adhesive spray (Tack Spray; Nitto Nitoms, Tokyo, Japan), and incubated with 5 μM Fluo-8 AM for 20 min. Equal volume of 200 μM cinnamaldehyde or l-menthol in HBSS containing 5 μM Fluo-8 AM was then gently added (the final concentration of the TRP agonists was 100 μM). The fluorescence of ciliated cells was observed under a Carl Zeiss Axioskop 2 Plus fluorescence microscope. The light source was an HBO 103 W/2 mercury vapor lamp. The light was let pass through a 475–495 nm bandpass filter for the excitation, and the emitted fluorescence was allowed to pass through a 515–565 nm bandpass filter. Fluorescence images were recorded just before and 5/10 min after the addition of the TRP agonist, using the high-speed high-sensitivity digital imaging system HAS-D71 (DITECT) attached to the microscope at a speed of 100 frames/sec. The fluorescence intensity was quantitatively analyzed by HAS-XViewer Camera Memory ver. 1.3.0.13 (DITECT). The fluorescence images were displayed in a 256-step arbitrary scale of 0 (no fluorescence) to 255 (most intense fluorescence) for each pixel of the images. The region of interest (ROI) that included a target cell was manually defined, and the mean pixel value of the ROI was calculated. The shape and size of the ROI were fixed for each target cell. The %fluorescence intensity was calculated for each cell by dividing the fluorescence intensity of interest by that just before the addition of the TRP agonist.

### Fluorescence immunohistochemistry

The specimens were fixed with 4% paraformaldehyde in 0.1 M phosphate buffer at pH 7.4 (PB) at 4 °C overnight. The fixed samples were transferred into a solution of 20% sucrose in 0.1 M phosphate-buffered saline at pH 7.4 (PBS) and incubated at 4 °C for 2 nights with 3–4 changes of solution. The samples were then frozen while embedded in Tissue-Tek OCT compound (Sakura Finetek, Tokyo, Japan) and stored at −80 °C until sectioning. Seven-μm-thick sections were prepared using a cryostat, mounted on silane-coated glass slides (Superfrost; Matsunami Glass Industries, Osaka, Japan), and air-dried. The sections were hydrated in PBS with 0.3% Triton X-100 (PBST) for 20 min and treated with 1.5% normal goat serum in PBST for 1 h. They were then incubated with rabbit anti-human TRPA1 polyclonal antibody (LS-B2819; LifeSpan BioSciences, Seattle, WA, USA) or rabbit anti-human TRPM8 polyclonal antibody (LS-C160235; LifeSpan BioSciences) at 4 °C overnight. Dilutions of the primary antibodies were 1:50 for TRPM8 and 1:200 for TRPA1, in PBST containing 0.5% bovine serum albumin (BSA). As a negative control, the primary antibodies were omitted from the process. After a brief rinse with PBST, the sections were reacted at room temperature for 2 h with a secondary antibody, Alexa Fluor 488-conjugated goat anti-rabbit IgG (Invitrogen, Eugene, OR, USA) diluted 1:1000 in PBST containing 0.5% BSA. The sections were coverslipped with Prolong Gold antifade reagent containing 4′,6‐diamidino‐2‐phenylindole dihydrochloride (DAPI; Invitrogen) and examined under a Carl Zeiss Axioskop 2 Plus fluorescence microscope. The light source was an HBO 103 W/2 mercury vapor lamp. The light passed through specific wavelength bandpass filters for excitation: 475–495 nm for Alexa Fluor 488 and 340–380 nm for DAPI. Similarly, the emitted fluorescence passed through a 515–565 nm bandpass filter for Alexa Fluor 488 and a 435–485 nm bandpass filter for DAPI. Images were captured using a Carl Zeiss AxioCam digital camera attached to the microscope.

### Preparation of total RNA

The collected tissues were minced with surgical scissors, soaked in 1 ml TRIzol Reagent (Invitrogen), and sonicated by an ultrasonic homogenizer (Taitec, Saitama, Japan). Two hundred μl chloroform was added, and after thorough shaking, the mixture was centrifuged at 22,000 xg for 15 min at 4 °C. The aqueous layer was transferred to another tube, and total RNA was extracted by the acid guanidiniumthiocyanate-phenol–chloroform method and cleaned up with a BioRobot EZ1 system (QIAGEN, Hilden, Germany), which enables fully automated extraction and purification of nucleic acids by magnetic bead technology. The purity of RNA was assessed by determining the ratio of light absorption at 260 nm (A_260_) to that at 280 nm (A_280_). An A_260_/A_280_ ratio in the 1.9–2.1 range was considered acceptable. The RNA concentration was determined from A_260_.

### Real-time reverse transcription-polymerase chain reaction (RT-PCR)

The total RNA was reverse-transcribed to cDNA with a High-Capacity RNA-to-cDNA Kit (Applied Biosystems, Foster City, CA, USA), which uses random primers. The real-time RT-PCR analysis was performed with an Applied Biosystems StepOnePlus real-time PCR system using TaqMan Fast Universal PCR Master Mix (Applied Biosystems) with *glyceraldehyde-3-phosphate dehydrogenase* (*GAPDH*) as a housekeeping gene according to the manufacturer's instructions. The TaqMan Gene Expression Assays for *TRPA1* (assay identification number: Hs00175798_m1), *TRPM8* (assay identification number: Hs01066596_m1), and *GAPDH* (assay identification number Hs99999905_m1) were purchased from Applied Biosystems. Ten ng cDNA in 1 µl was mixed with TaqMan Universal PCR Master Mix with AmpErase (uracil N-glycosylase) and the primer/probe set of the TaqMan Gene Expression Assays, and the mixture was subjected to PCR amplification with real-time detection. The thermal cycler conditions were as follows: holding at 95 °C for 2 min, followed by 40 cycles of a two-step polymerase chain reaction of 95 °C for 1 sec and 60 °C for 20 sec. Each sample was assayed in duplicate. The measured threshold cycle (C_T_) was normalized by subtracting the C_T_ for *GAPDH* of each sample from those for *TRPA1* and *TRPM8*. From the obtained ∆C_T_, the ratio of the target mRNA to *GAPDH* mRNA was calculated by the following formula:

Target mRNA/*GAPDH* mRNA ratio = $2^{{-\Delta \text C}_\text T}$.

### Statistical analysis

Data are expressed as means±SEM. Statistical analysis was performed with the BellCurve for Excel Statistics (Social Survey Research Information Co., Tokyo, Japan). Differences between two groups were analyzed by a two-tailed paired *t*-test. *P*-values < 0.05 were considered significant.

## Results

**Fig. 1:**
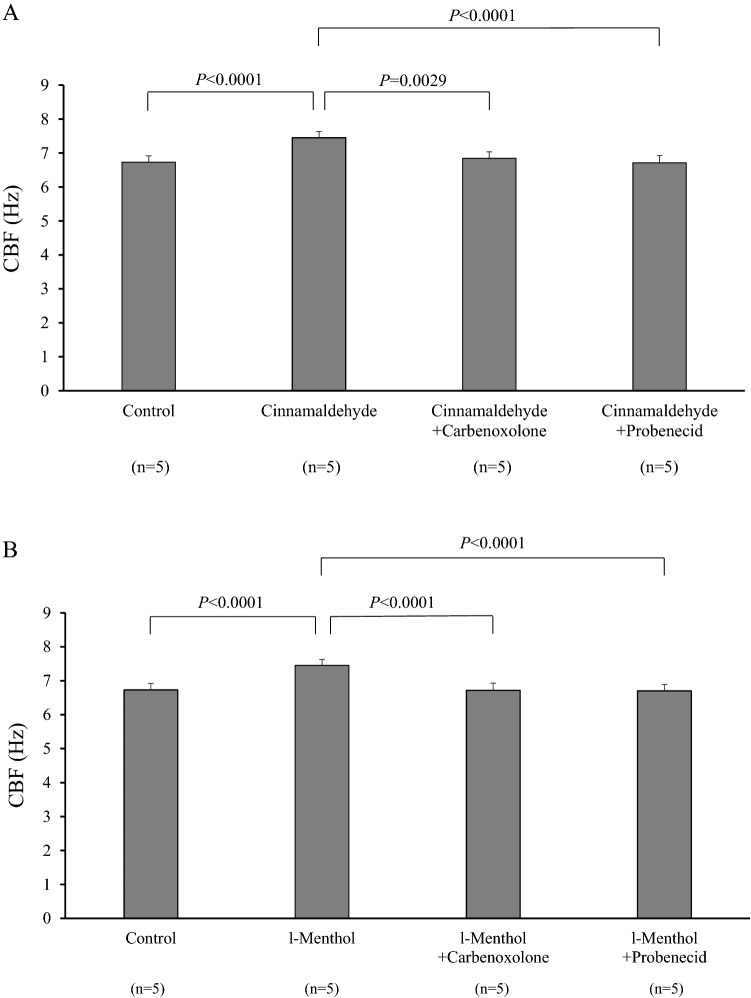
Effects of TRPA1 and TRPM8 agonists on CBF with or without pannexin-1 blockers. The preincubation time was 5 min for cinnamaldehyde and 10 min for l-menthol. CBF was significantly increased by the addition of either (**A**) cinnamaldehyde (100 μM) or (**B**) l-menthol (100 μM). These increases were inhibited by simultaneous loading of a pannexin-1 blocker, either carbenoxolone (10 μM) or probenecid (300 μM)

Figures 1A and B show the effects of cinnamaldehyde (100 μM) and l-menthol (100 μM) on CBF, respectively. The preincubation time was 5 min for cinnamaldehyde and 10 min for l-menthol. The CBF was rapidly and significantly increased after the addition of the TRP agonists, reached a plateau within 5 and 10 min, respectively, and was kept elevated at least 30 min under the presence of the agonists. These increases were inhibited by simultaneous loading of a pannexin-1 blocker, either carbenoxolone (10 μM) or probenecid (300 μM).

**Fig. 2:**
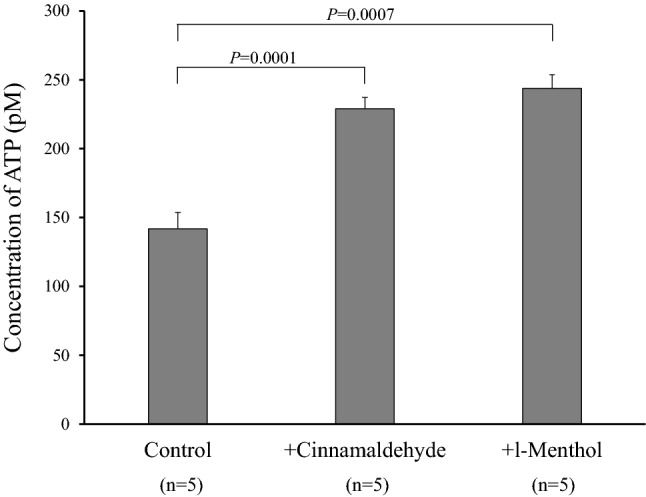
Effects of cinnamaldehyde and l-menthol on the ATP release from the turbinate mucosa. Both agonists significantly increased the ATP release at concentrations of 100 μM

Figure 2 represents the effects of cinnamaldehyde (100 μM) and l-menthol (100 μM) on the ATP release from the turbinate mucosa. The incubation time was 5 min for cinnamaldehyde and 10 min for l-menthol. Both agonists significantly increased the ATP release.

**Fig. 3:**
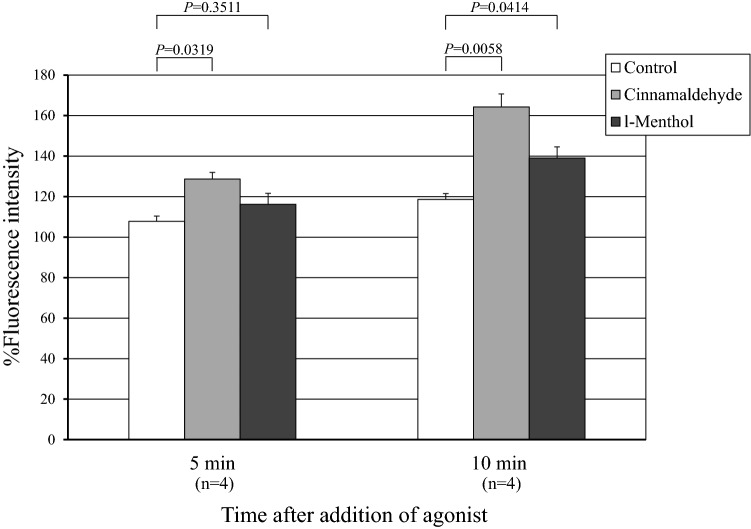
Changes in Fluo-8 fluorescence of isolated ciliated cells after the addition of TRP agonists. %Fluorescence intensity was significantly increased 5 and 10 min after the addition of cinnamaldehyde (100 μM) in a time-dependent manner. %Fluorescence intensity was also significantly increased 10 min after the addition of l-menthol (100 μM)

Figure 3 shows changes in Fluo-8 fluorescence of isolated ciliated cells after the addition of the TRP agonists. %Fluorescence intensity was significantly increased 5 and 10 min after the addition of cinnamaldehyde (100 μM) in a time-dependent manner. %Fluorescence intensity was also significantly increased 10 min after the addition of l-menthol (100 μM).

**Fig. 4:**
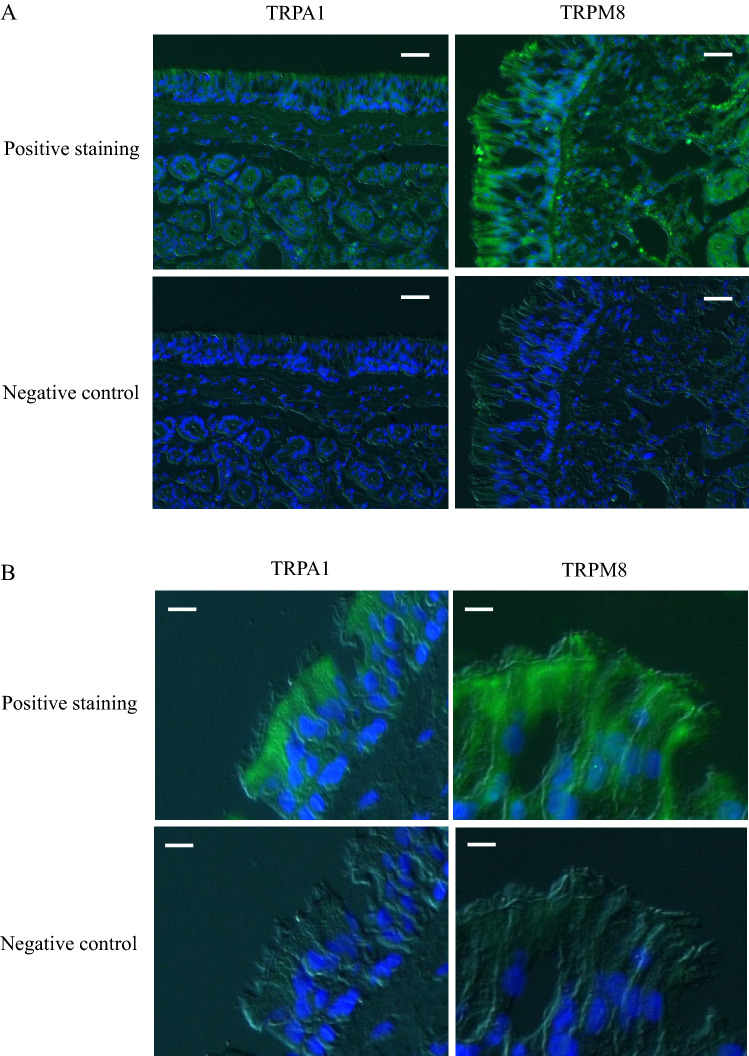
Representative photomicrographs of fluorescence immunohistochemical staining of the turbinate mucosa for TRPA1 and TRPM8. Green and blue colors express the fluorescence of Alexa Fluor 488 and DAPI, respectively. (**A**) Fluorescence is observed on the apical surface of the epithelial cells and in the submucosal nasal glands for both TRPA1 and TRPM8. Fluorescence for TRPA1 is weak, while that for TRPM8 is moderate. Scale bar = 20 μM. (**B**) Higher magnification shows that the cilia of the epithelial cells exhibit immunoreactivity for TRPA1 and TRPM8. Scale bar = 5 μM. (Color figure online)

Figure 4 displays representative photomicrographs of fluorescence immunohistochemical staining of the turbinate mucosa for TRPA1 and TRPM8. Fluorescence was observed on the apical surface of the epithelial cells and in the submucosal nasal glands for both TRPA1 and TRPM8 (Fig. 4A). Fluorescence for TRPA1 was weak while that for TRPM8 was moderate (Fig. 4A). Observation at higher magnification revealed that the cilia of the epithelial cells exhibited immunoreactivity for TRPA1 and TRPM8 (Fig. 4B).

**Fig. 5:**
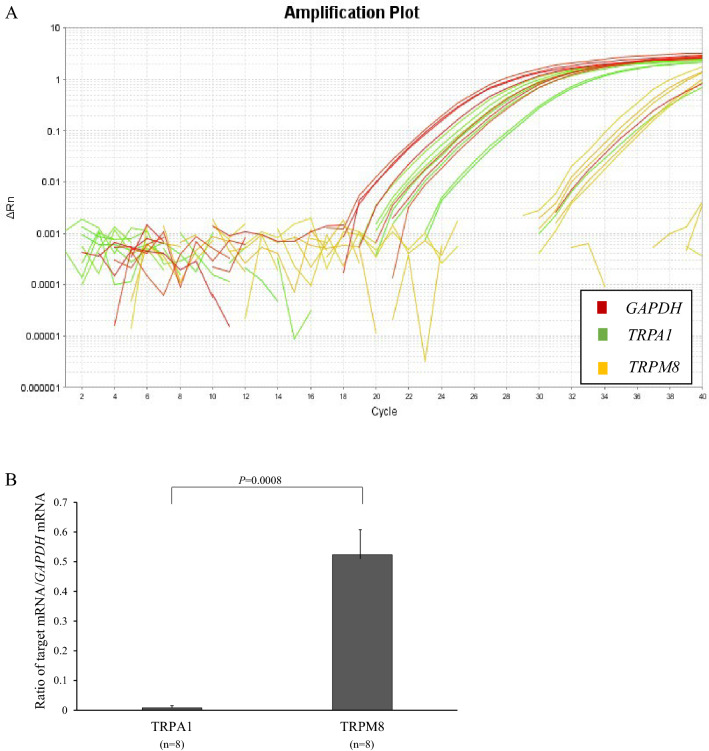
Real-time RT-PCR for *TRPA1/M8*. (**A**) Representative trace of amplification plots. The exponential rise of the trace proves the presence of *TRPA1* and *TRPM8* mRNAs in the human nasal mucosa. (**B**) Expression levels of *TRPA1/M8* mRNA. The expression level of *TRPM8* mRNA was significantly higher than that of *TRPA1* mRNA (*P* = 0.0008)

Results of real-time RT-PCR are presented in Fig. 5. The exponential rise of the trace of amplification plots (Fig. 5A) proved the presence of *TRPA1* mRNA and *TRPM8* mRNA in the human nasal mucosa. The average ratios of *TRPA1* mRNA/*GAPDH* mRNA and *TRPM8* mRNA/*GAPDH* mRNA were 0.0080±0.0076 and 0.5235±0.0968, respectively (n = 8; Fig. 5B). The expression level of *TRPM8* mRNA was significantly higher than that of *TRPA1* mRNA (*P* = 0.0008).

These results indicate that TRPA1 and TRPM8 are expressed in the nasal mucosa and participate in the regulation of ciliary beating, and that this regulatory action is mediated by the pannexin-1 channel.

## Discussion

In the present study, we showed that agonists of TRPA1 and TRPM8 upregulated ciliary beating and that these stimulatory effects were inhibited by pannexin-1 blockers. These TRPA1/M8 agonists also increased the ATP release from the nasal mucosa and [Ca^2+^]_i_ of isolated ciliated cells. We further demonstrated that both TRPA1 and TRPM8 are expressed in the nasal mucosa at the protein and transcriptional levels.

The TRP channels constitute a large family of nonselective cation channels that respond to a variety of chemical and physical stimuli, such as pH, light, mechanical stress, temperature, osmolality and chemical irritants, and participate in the processes of mechano reception, thermosensation, and nociception. The TRP channels are classified into 7 subfamilies according to similarities of amino acid sequences: TRPV, TRPM, TRPA, TRPC, TRPP, TRPML, and TRPN. The last subfamily, TRPN, is present in invertebrates and some vertebrates but absent in mammals [11].

Perception of temperature is a crucial function for the survival of life. From the 1990s to the 2000s, thermosensitive receptors were discovered to be TRPs; that is, TRPV1, TRPV2, TRPV3, TRPV4, TRPM2, TRPM3, TRPM4, TRPM5, TRPM8, TRPA1, and TRPC5 in mammalian species [12]. Among these thermosensitive TRPs, it has been established that TRPV1 and 2 respond to hot temperatures, TRPV3 and TRPV4 respond to warm temperatures, and TRPM8 and TRPA1 respond to cool/cold temperatures [6].

The temperature for TRPM8 activation is 25–28 °C and lower, while that for TRPA1 activation is < 17 °C [6]. As shown in the present study, both channels are expressed in the human nasal mucosa. End-inspiratory nasal mucosal temperature typically drops below 30 °C [13, 14], leading to the activation of TRPM8. Because of this, TRPM8 is thought to be a nasal airflow sensor [3, 4]. On the other hand, TRPA1 does not respond unless the temperature drops below 20 °C and probably acts as a receptor for pain and itching rather than as an airflow sensor [15, 16].

Normal nasal breathing is essential to maintain respiratory health [1, 2]. A narrowed nasal airway and the consequent increase in nasal airway resistance augment the negative pressure of the pharyngeal space in inhalation, leading to collapse of the oropharynx particularly during sleep. Blockage of the nasal airway causes mouth breathing followed by a posterior shift of the base of the tongue and collapse of the pharyngeal space. Further, the sensation of nasal obstruction can induce mental and physical disorders, such as impaired concentration, drowsiness, fatigue, headache, sleep disturbance, and even deterioration of quality of life [1, 2].

Ciliary beating, an essential function for mucosal defense in the respiratory tract, is suppressed as the mucosal temperature falls. Clary-Meinesz et al. [5] measured the ex vivo CBF of ciliated cells of the nasal and tracheal mucosae while changing the culture temperature from 50 to 5 °C. They found that CBF was gradually decreased until the temperature fell to 20 °C, but the gradient of decrease became steeper below 20 °C. This observation suggests that ciliary beating is controlled differently between the two temperature ranges. The present results imply that activation of TRPM8 exerts a compensatory effect to keep ciliary beating at a stable level despite decreases in temperature. This effect is advantageous for preserving airway mucosal defense while nasal breathing cools the mucosa. However, at temperatures under 20 °C, the compensatory effect of TRPA1/TRPM8 may be overwhelmed by the inhibitory effect of the low temperatures.

Our results also indicated that the stimulatory effects of the TRPA1/TRPM8 agonists are mediated by pannexin-1, which is considered an ATP-releasing channel. Pannexins were originally cloned as gap junction-related proteins. In reality, they are transmembrane hemichannels rather than gap junction proteins. Pannexins has three isoforms, pannexin-1, pannexin-2, and pannexin-3. It has been reported that pannexin-1 releases ATP extracellularly in response to various stimuli, such as membrane depolarization, hypotonicity, mechanical stretch/strain, elevated extracellular K^+^, and cleavage of the intracellular domain by caspase [17]. According to our previous study, pannexin-1 is a key component in the regulatory pathway of the ciliary beating of the nasal mucosa [18]. Because the TRP channels, including TRPA1 and TRPM8, are cation channels, activation of these channels lets cations, such as Ca^2+^, Na^+^, and Mg^2+^, through into the cell and elicits depolarization of the membrane potential. Thereby, the pannexin-1 channel opens, leading to the extracellular ATP release and consequent upregulation of ciliary beating. In addition, our recent study revealed that the T-type voltage-gated Ca^2+^ channel exists in the cilia of the nasal mucosa and participates in the regulation of ciliary beating [10]. This channel should be activated by membrane depolarization and may contribute to the upregulation of ciliary beating induced by the TRPA1/M8 agonists.

There are some limitations in this study. The CBF and ATP release were measured, and [Ca^2+^]_i_ imaging was performed at room temperature, approximately 24 °C. Because TRPM8 is thought to be activated at this temperature, l-menthol loading inevitably results in double stimulation of TRPM8. To avoid this confusion, it is ideal to perform l-menthol-loading experiments at a temperature that does not activate TRPM8, such as ≥ 30 °C. This is one of the weak points of the present study. However, because all experiments were performed at the same temperature, data bias caused by the temperature effect is considered the same in each experiment. Therefore, it is meaningful to examine the effect of the agonist of a thermoreceptor at a fixed temperature, even if the temperature activates the thermoreceptor.

## Conclusions

We investigated the expressions of the cool/cold thermoreceptors, TRPA1 and TRPM8, in the nasal mucosa and the effects of the specific agonists of these receptors on ciliary beating. Both receptors were expressed at the protein and transcriptional levels. Stimulation of these receptors by specific agonists significantly upregulated ciliary beating, promoted ATP release from the mucosa, and increased [Ca^2+^]_i_ of isolated ciliated cells. This effect is advantageous for maintaining both airway mucosal defense and normal nasal breathing simultaneously. The stimulatory effects of the TRPA1/TRPM8 agonists were inhibited by pannexin-1 blockers, indicating that pannexin-1 activation and probably ATP release occur after TRPA1/TRPM8 activation. Understanding the regulatory mechanism of airway ciliary beating should be beneficial for developing new, effective treatments for intractable upper and lower airway diseases.
